# Gel versus capillary electrophoresis genotyping for categorizing treatment outcomes in two anti-malarial trials in Uganda

**DOI:** 10.1186/1475-2875-9-19

**Published:** 2010-01-15

**Authors:** Vinay Gupta, Grant Dorsey, Alan E Hubbard, Philip J Rosenthal, Bryan Greenhouse

**Affiliations:** 1 Department of Medicine, University of California, San Francisco, Box 0811, CA 94143, USA; 2 Columbia University, College of Physicians and Surgeons, 630 West 168th Street, New York, NY 10032, USA; 3 Division of Biostatistics, University of California, Berkeley, 101 Haviland Hall, Berkeley, CA 94720, USA

## Abstract

**Background:**

Molecular genotyping is performed in anti-malarial trials to determine whether recurrent parasitaemia after therapy represents a recrudescence (treatment failure) or new infection. The use of capillary instead of agarose gel electrophoresis for genotyping offers technical advantages, but it is unclear whether capillary electrophoresis will result in improved classification of anti-malarial treatment outcomes.

**Methods:**

Samples were genotyped using both gel and capillary electrophoresis from randomized trials of artemether-lumefantrine (AL) vs. dihydroartemisinin-piperaquine (DP) performed in two areas of Uganda: Kanungu, where transmission is moderate, and Apac, where transmission is very high. Both gel and capillary methods evaluated polymorphic regions of the merozoite surface protein 1 and 2 and glutamine rich protein genes.

**Results:**

Capillary electrophoresis detected more alleles and provided higher discriminatory power than agarose gel electrophoresis at both study sites. There was only moderate agreement between classification of outcomes with the two methods in Kanungu (kappa = 0.66) and poor agreement in Apac (kappa = 0.24). Overall efficacy results were similar when using gel vs. capillary methods in Kanungu (42-day risk of treatment failure for AL: 6.9% vs. 5.5%, p = 0.4; DP 2.4% vs. 2.9%, p = 0.5). However, the measured risk of recrudescence was significantly higher when using gel vs. capillary electrophoresis in Apac (risk of treatment failure for AL: 17.0% vs. 10.7%, p = 0.02; DP: 8.5% vs. 3.4%, p = 0.03). Risk differences between AL and DP were not significantly different whether gel or capillary methods were used.

**Conclusions:**

Genotyping with gel electrophoresis overestimates the risk of recrudescence in anti-malarial trials performed in areas of high transmission intensity. Capillary electrophoresis provides more accurate outcomes for such trials and should be performed when possible. In areas of moderate transmission, gel electrophoresis appears adequate to estimate comparative risks of treatment failure.

## Background

When anti-malarial drug efficacy trials are performed in malaria endemic areas, molecular genotyping is required to determine whether recurrent parasitaemia after therapy represents a recrudescence (treatment failure) or new infection [1]. Most commonly, genotyping methods take advantage of variation in highly polymorphic genes of *Plasmodium falciparum*, including merozoite surface proteins 1 and 2(*msp1 *and *msp2*) and glutamine-rich protein (*glurp*) [2]. To discriminate *P. falciparum *strains, the sizes of amplified products of these genes are compared, routinely based on migration patterns with agarose-gel electrophoresis because of ease of use and low cost. However, gel electrophoresis may not provide adequate discrimination of alleles, especially in high transmission settings, where high multiplicity of infection (MOI) can lead to a large number of genotyping misclassifications due either to missed detection of alleles or to alleles matching by chance [3,4]. When the probability that alleles will match by chance is high, genotyping-adjusted estimates of the risk of treatment failure may be grossly overestimated [3,5]. Obtaining accurate estimates of the risk of treatment failure is important for guiding policy, as decisions regarding anti-malarial therapy are primarily based on these estimates.

In response to limitations in standard genotyping methods, the World Health Organization (WHO) recently recommended genotyping with capillary electrophoresis, where possible, to increase test sensitivity and discriminatory power [6]. Capillary electrophoresis was recommended due to technical advantages over agarose gel electrophoresis, most importantly the ability to measure the size of PCR products with very high resolution. In fact, a recent study found that capillary electrophoresis detected more *msp1 *and *msp2 *alleles with more precise sizing compared to gel electrophoresis [7]. However, in an anti-malarial treatment trial, this same study demonstrated that genotyping using *msp1 *and *msp2 *with either gel or capillary electrophoresis provided identical outcome classifications [7].

Capillary electrophoresis requires expensive equipment and is not as readily available as gel electrophoresis. At present, it remains unclear whether capillary electrophoresis will, in practice, result in improved classification of anti-malarial treatment outcomes compared to gel electrophoresis and thus whether the added expense of this methodology is warranted. To critically assess gel and capillary electrophoresis-based genotyping, the two methods were directly compared with analysis of *msp2*, *glurp*, and *msp1 *alleles in clinical samples. Samples were used from two randomized trials comparing the anti-malarial efficacy of artemether-lumefantrine (AL) and dihydroartemisinin-piperaquine (DP) at sites with very different malaria transmission intensities.

## Methods

### Study sites and trial design

Clinical samples were utilized from trials conducted at two sites with markedly different transmission intensities, Kihihi Health Centre in Kanungu District in western Uganda and Aduku Health Centre in Apac District in central Uganda. In Kanungu, malaria is mesoendemic, with an entomological inoculation rate (EIR) estimated at seven infectious bites per person per year [8]. In contrast, in Apac, malaria is holoendemic, with an EIR estimated at 1,564 infectious bites per person per year [8].

The details of the clinical trials have been published elsewhere [5,9]. In both trials, children aged 6 months to 10 years with uncomplicated falciparum malaria were randomized to receive either AL or DP and followed for 42 days. Treatment outcomes were assessed according to WHO guidelines [10]. All subjects with recurrent parasitaemia detected between 7 and 42 days after the initiation of therapy had samples genotyped from the day of treatment (Day 0) and day of identification of recurrent parasitaemia (day of failure or Day F). Genotyping was performed in a stepwise fashion using *msp2*, *glurp*, and then *msp1 *according to WHO guidelines [6], with analysis by capillary and gel electrophoresis in parallel. As discussed in these guidelines, loci with highest diversity should be used first. For each locus, if genotyping assigned an outcome as a new infection (no shared alleles between days of initial and recurrent parasitaemia), no further loci were studied; if genotyping assigned an outcome as a potential recrudescence (at least one shared allele), additional loci were assessed, in the order *msp2*, then *glurp*, then *msp1*. Sample pairs which shared at least one allele at every locus successfully genotyped were defined as a recrudescence. Those samples that failed genotyping with *msp2 *underwent PCR-based speciation to distinguish between *P. falciparum *and other plasmodial species. Samples containing only non-falciparum DNA were not genotyped further. If a sample failed genotyping with all three markers, it was categorized as having failed molecular genotyping.

### Genotyping using agarose gel electrophoresis

Amplification of the polymorphic surface antigens *msp2*, *glurp*, and *msp1 *was performed according to standard WHO protocols [6]. In brief, DNA was extracted from stored blood spots on filter paper into 130 μl of water using a standard Chelex technique [11] and 2 μl of template DNA was amplified using nested PCR. PCR was performed in a Bio-Rad C-1000 thermal cycler (Bio-Rad Laboratories, Inc, Hercules, CA USA). Primers corresponding to allelic families for *msp2 *(FC27 and IC3D7) and *msp1 *(K1, MAD20, and RO33) and a polymorphic region of *glurp *were used as previously described [12,13]. The resulting PCR products were separated on a 2.5% agarose gel (UltraPure Agarose: Invitrogen, Carlsbad, CA) stained with ethidium bromide. A technician blinded to the identity of the samples estimated the size of PCR products using GelCompar II software (Applied Maths, Sint-Martens-Latem, Belgium). Alleles in paired samples were considered a match for *msp2 *and *msp1 *if within 10 base pairs and for *glurp *if within 20 base pairs.

### Genotyping using capillary electrophoresis

For capillary electrophoresis, reaction and cycling conditions were optimized based on previously published protocols before conditions were chosen (primer sequences, cycling and reaction conditions in Additional File 1, Table S1). All nested reactions had forward primers labelled with fluorophores at the 5' end and reverse primers tagged with a 5' GTGTCTT "tail" to promote addition of an extra adenosine base for more uniform PCR product sizes [14]. All fluorescent primers except for the K1 family-specific primer for *msp1 *used non-proprietary HEX or 6-FAM labels to minimize cost.

After amplification, the PCR products were prepared for analysis by mixing 2 μl of 1:10 diluted sample with 10 μl Hi-Di formamide and 0.2 μl of the appropriate size standard (Genescan 500HD ROX (Applied Biosystems) for *msp1 *and *msp2*; Genescan 1000HD ROX (Applied Biosystems) for *glurp*. Samples were then denatured at 95°C for 5 minutes and run on an Applied Biosystems 3730xl DNA Analyzer. Alleles were sized with GeneMapper 4.0 software (Applied Biosystems). Alleles in paired samples were considered a match for *msp2 *and *msp1 *if within 1 base. Based on our experience showing less accurate sizing for larger fragments, alleles for *glurp *were considered a match if within 1 base for alleles <880 bases, 1.5 bases for alleles between 880 and 930, 2 bases for alleles between 930 and 980, 2.5 bases for alleles between 980 and 1030, and 3 bases for alleles between 1030 and 1100.

### Statistical analysis

Statistical analysis was performed using Stata SE version 10 (StataCorp., College Station, Texas) and R version 2.9.0 (R Foundation for Statistical Computing, Vienna, Austria). For each locus, the empiric distribution of allele sizes detected on Day 0 was used to estimate the probability that two randomly selected alleles will match by chance without arbitrary binning of alleles. For every Day 0 allele detected, the proportion of other detected alleles that met the appropriate match criteria (e.g. within 10 base pairs for MSP2 gel and 1 base for MSP2 capillary electrophoresis) was calculated and used to estimate the probability that the particular Day 0 allele would match another by chance. For example, if 1,000 MSP2 gel alleles were detected in 500 samples, and allele "A" falls within 10 base pairs in size of 100 of the other alleles, the probability of that particular allele matching another by chance is 100/1,000 or 0.1. The mean of these proportions over all Day 0 alleles was then used to estimate the overall probability of a match occurring by chance between two randomly selected alleles. Of note, two alleles within an allelic family may match based on size but have different sequences; from a misclassification standpoint it does not matter whether two different parasites share a matching allele which differs based on sequence or is identical in sequence.

The probability of a match (P_match_) occurring between the Day 0 and Day F samples, which may both contain multiple alleles, was calculated by taking into account the actual alleles present in the Day 0 sample as well as the number of alleles present in the Day F sample (*n*). This probability has previously been calculated after first assigning each allele to a bin based on size [3]. However, arbitrary binning of alleles does not accurately reflect the probability of calling alleles a match in practice, e.g. alleles of size 299 and 300 may fall into different bins but would be called a match in practice. Therefore, a slightly different method was used as follows, which gives similar results but is less biased, less computationally intensive, and easier to implement. For each sample pair, 10,000 possible Day F samples were simulated by randomly selecting combinations of *n *alleles from the empiric distribution of Day 0 alleles. The proportion of these combinations that met the criteria for a match with the actual Day 0 sample (at least one allele matching) was then calculated and used to estimate P_match _for that sample pair.

A bootstrap with 10,000 repetitions was used to test the hypothesis that the probability of two alleles matching by chance was the same between gel and capillary electrophoresis. Given that there were *i *subjects, in each bootstrap repetition all the alleles from *i *Day 0 subjects from both gel and capillary electrophoresis were sampled with replacement. The probability of two alleles matching by chance for both gel and capillary electrophoresis was then calculated from the bootstrap sample as described above, and the difference between these two probabilities was calculated. The Wilcoxon Rank Sum test was used for comparisons of other characteristics between gel and capillary electrophoresis. A kappa statistic was used to test inter-rater agreement between gel and capillary electrophoresis results. The 42-day risk of treatment failure was defined as recurrent malaria due to recrudescent parasites as determined by genotyping. Risk of failure was estimated using the Kaplan-Meier product limit formula. Data were censored for subjects who did not complete 42 days of follow-up and for new infections. A bootstrap with 1,000 repetitions was used to test the hypothesis that the 42-day risk of failure was different between groups.

## Results

### Samples genotyped

**Figure 1:**
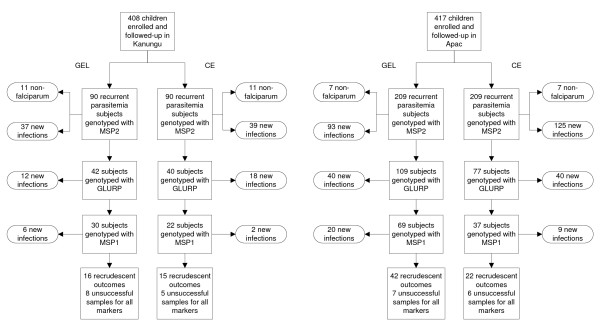
**Genotyping using markers sequentially at both study sites**. Subjects with recurrent parasitaemia in Kanungu and Apac had samples genotyped in a stepwise fashion in the order shown. A new infection was defined as no alleles matching in samples taken on the day of treatment and the day of recurrent parasitaemia. A subject not classified as a new infection by one marker was then genotyped sequentially with the next marker. Those not classified as having a new infection after being genotyped by all three markers were classified as having a recrudescence, as long as genotyping was successful for at least one marker.

To directly compare the results of gel versus capillary electrophoresis, the samples from two drug efficacy trials were independently genotyped with each method, using the stepwise algorithm of assessing *msp2*, *glurp*, then *msp1 *recommended by the WHO [6]. In Kanungu, a region of moderate transmission intensity, 212 children were randomized to treatment with DP, of whom 26 (12%) required genotyping, and 196 were treated with AL, of whom 64 (33%) required genotyping (Figure 1). In Apac, a region of very high transmission intensity, 211 children were randomized to treatment with DP, of whom 92 (44%) required genotyping, and 210 were treated with AL, of whom 119 (57%) required genotyping.

### Discriminatory ability of gel and capillary electrophoresis

**Figure 2:**
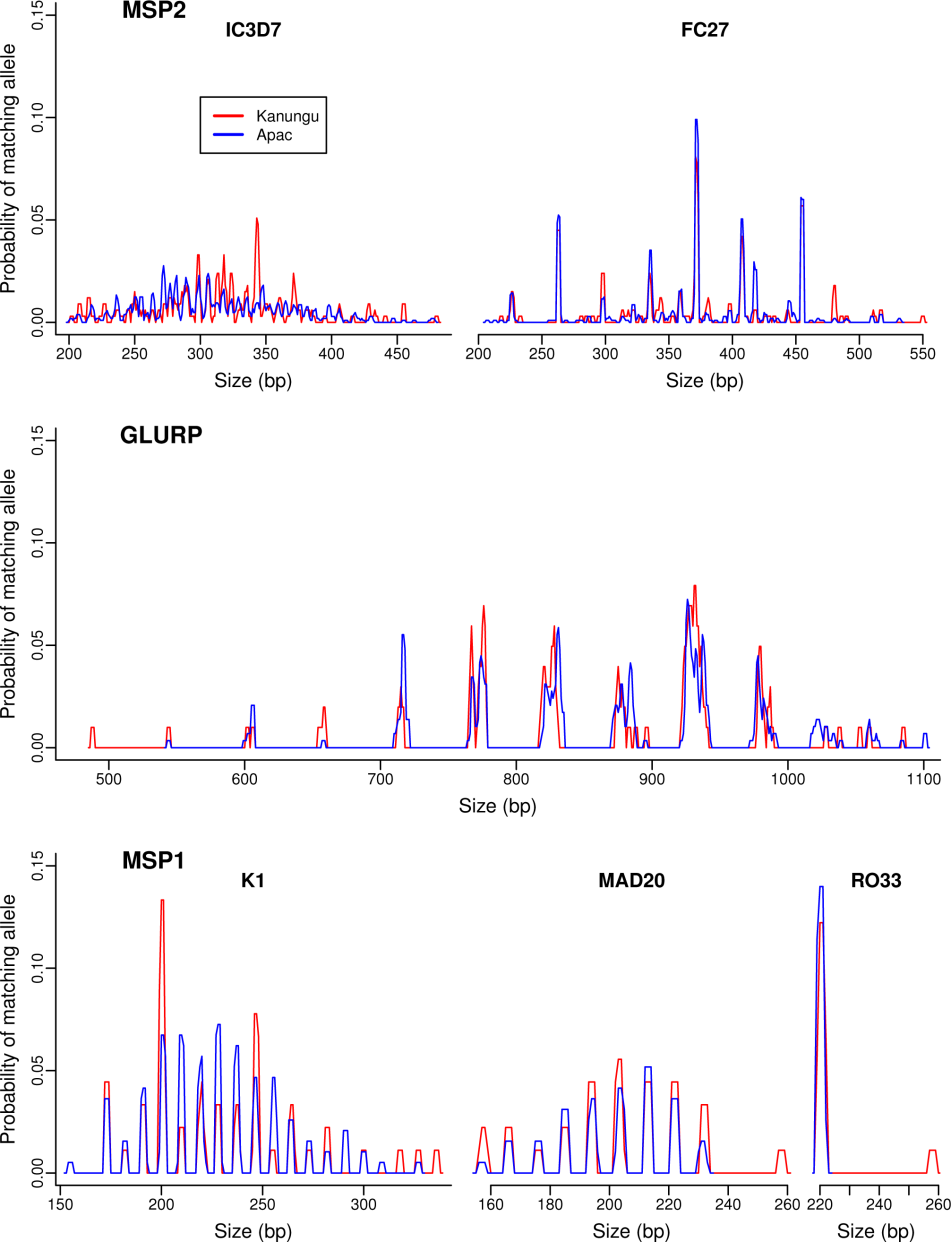
**Allele sizes measured by capillary electrophoresis at both study sites**. The y axis represents the probability of an allele of the given size matching another by chance. As described in the methods, this is calculated as the proportion of all allele measurements from all Day 0 samples that fall within the specified match criteria (within 1 base in size for *msp1 *and *msp2*, greater for *glurp *alleles 880 bases or larger, see methods for more detail).

Based on the same criteria used to distinguish alleles in sample pairs, two *msp2 *alleles picked at random were 2-3 times more likely to match using gel versus capillary electrophoresis due to the greater ability of capillary electrophoresis to discriminate alleles (Table 1). The probability of two *msp2 *alleles matching was very similar between the sites, indicating a similar degree of genetic diversity in parasites from each site (Table 1, Figure 2). Similar relationships were seen for *glurp *and *msp1 *with *glurp *alleles five times more likely and *msp1 *alleles twice more likely to match by chance with gel compared to capillary electrophoresis at both sites. However, a direct comparison between methods can only be made with *msp2*, since different sample pairs were genotyped with gel and capillary electrophoresis at *glurp *and *msp1 *in the stepwise algorithm.

**Table 1:** Characteristics of genotyping *msp2 *using agarose gel versus capillary electrophoresis.

Study Location	Characteristic	Gel^a^	CE^b^	P value^c^
Kanungu (n = 90)	Probability two randomly selected alleles will match by chance^d^	0.066	0.025	<0.001
	MOI Day 0^e^, mean (SD)	2.73 (1.53)	3.82 (2.58)	<0.001
	MOI Day F^f^, mean (SD)	1.23 (1.14)	2.02 (1.82)	<0.001
	P_match _^g^, median (IQR)	0.20 (0.11 - 0.36)	0.16 (0.06 - 0.26)	0.001
Apac (n = 209)	Probability two randomly selected alleles will match	0.061	0.028	<0.001
	MOI Day 0, mean (SD)	3.65 (1.80)	5.06 (3.42)	<0.001
	MOI Day F, mean (SD)	2.42 (1.59)	3.26 (2.62)	<0.001
	P_match_, median (IQR)	0.41 (0.23 - 0.59)	0.27 (0.12 - 0.50)	<0.001

^a ^Gel, Agarose gel electrophoresis

^b ^CE, Capillary electrophoresis

^c ^P values testing the hypothesis Gel is different than CE using a bootstrap test for the probability two alleles will match by chance, and the Wilcoxon Rank Sum test for other characteristics

^d ^Calculated using the empiric distribution of Day 0 alleles and criteria for a match based on the electrophoresis method

^e ^Multiplicity of infection (number of alleles detected) on Day 0 (day of anti-malarial treatment)

^f ^Multiplicity of infection on Day F (day of failure, i.e. recurrent parasitaemia)

^g ^Probability of a genotyping match occurring by chance for each sample, calculated using the actual alleles present on Day 0 and the multiplicity of infection on Day F (MOI-F). Ten thousand random combinations of MOI-F alleles were chosen for Day F using the empiric frequency distribution, and P_match _is the proportion of these combinations that had at least one allele match with the Day 0 alleles.

The ability to determine whether a pair of samples represents a recrudescence or new infection is influenced by the MOI (multiplicity of infection), or number of strains detected in each sample, as the probability of having at least one allele match by chance between the two samples (P_match_) [3] increases as MOI increases. In addition, the risk of not detecting a minority allele also increases as MOI increases, raising the probability of a recrudescence being misclassified as a new infection. At both sites, significantly more *msp2 *alleles were detected using capillary than gel electrophoresis (Table 1). Despite the higher MOI identified by capillary electrophoresis, this technique yielded a significantly lower P_match _than gel electrophoresis due to its higher resolution. Similar results were seen for *glurp *and *msp1*, with capillary electrophoresis showing a higher MOI and lower P_match _than gel electrophoresis. These data suggest that genotyping misclassification of both recrudescences and new infections may be lower with capillary than gel electrophoresis.

Comparing the two trial sites, MOI was higher in Apac than Kanungu (Table 1). As expected, given the similar genetic diversity at both sites, P_match _was higher in Apac for both gel and capillary methods (p < 0.001 for both comparisons). Thus, misclassification of new infections as recrudescences is more likely in Apac than Kanungu.

### Agreement between gel and capillary electrophoresis results

When directly comparing the final genotyping results from gel and capillary electrophoresis algorithms, moderate agreement was observed (kappa = 0.66) in Kanungu (Table 2). However, only 11 of 19 (58%) sample pairs classified as a recrudescence by at least one method were in agreement (Table 2). At this site, discordant results occurred approximately equally in both directions (five recrudescences by gel, three recrudescences by capillary). Fewer genotyping failures were observed when using the capillary protocol (five failures) compared to gel electrophoresis (eight failures); all five capillary genotyping failures were also failures by the gel protocol.

**Table 2:** Agreement between gel and capillary electrophoresis results

Study Location	Capillary electrophoresis result	Gel Electrophoresis result			
		New Infection	Recrudescence	Genotyping Failure	Total:
Kanungu	New Infection	52	5	2	59
	Recrudescence	3	11	1	15
	Genotyping Failure	0	0	5	5
	Total:	55	16	8	79
Apac	New Infection	142	31	1	174
	Recrudescence	11	11	0	22
	Genotyping Failure	0	0	6	6

	Total:	153	42	7	202

In Apac, gel and capillary results were more discordant (kappa = 0.24), with only 11 of 53 (21%) sample pairs classified as a recrudescence by at least one method in agreement. At this site, most of the discordant results were classified as recrudescences by gel and new infections by capillary electrophoresis (31 of 42 discordant results, Table 2). To better understand why such a high degree of discordance was observed in Apac, the alleles detected using both methods were analysed in all discordant samples. In 16 of the 31 (52%) discordant sample pairs classified as recrudescences by gel electrophoresis, the alleles classified as recrudescent had analogous alleles detected by the capillary method. However, these alleles were sufficiently different in size to be beyond the more stringent match criteria for capillary electrophoresis and were thus classified as new infections. In the remaining 15 sample pairs, the alleles classified as recrudescent by gel electrophoresis did not have analogous alleles detected by the capillary method. In all 11 discordant sample pairs classified as recrudescences by capillary electrophoresis, the alleles classified as recrudescent by this method did not have analogous alleles detected by the gel method. Thus, discordance in results due to missed alleles by one method or generation of artefact in the other occurred similarly in both directions, while the more stringent match criteria of capillary electrophoresis largely drove the higher number of discordant results, which were classified as recrudescences by gel electrophoresis.

### Genotyping-adjusted clinical trial results using gel and capillary electrophoresis

The analysis was completed by evaluating the results of the anti-malarial trials using both genotyping modalities. In Kanungu, a few more sample pairs were classified as new infections early in the algorithm by capillary electrophoresis (Figure 1), but after all three loci were assessed, the measured 42-day risks of treatment failure for AL and DP were similar whether outcomes were classified based on genotyping by gel or capillary electrophoresis (Table 3). In contrast, in Apac, overall results for gel and capillary electrophoresis remained markedly different, with twice as many outcomes classified as recrudescent with the gel electrophoresis methodology. The measured 42-day risks of treatment failure for both AL and DP were significantly higher when genotyped using gel rather than capillary electrophoresis. However, the risk differences between AL and DP were similar with both genotyping methods. Thus, in Kanungu genotyping with either method led to similar measures of treatment outcome, while in Apac the two methods led to significant differences in absolute, but not comparative, results for the two treatment arms.

**Table 3:** Results of clinical trials by genotyping modality

Study Location	Treatment Arm	Genotyping-corrected 42-day Risk of Treatment Failure, % (95% CI)		**Risk Difference**, Gel vs. CE, % (p value)
		**Gel**	**CE**	
Kanungu	AL	6.9 (3.8-12.1)	5.5 (2.9-10.3)	1.4 (0.4)
	DP	2.4 (1.0-5.7)	2.9 (1.3-6.3)	-0.5 (0.5)
Risk Difference, AL vs. DP, % (p value)		4.5 (0.07)	2.6 (0.3)	
Apac	AL	17.0 (11.8-24.0)	10.7 (6.6-17.1)	6.3 (0.02)
	DP	8.5 (5.2-13.7)	3.4 (1.5-7.4)	5.1 (0.03)
Risk Difference, AL vs. DP, % (p value)		8.5 (0.03)	7.3 (0.02)	

Risks of treatment failure calculated using the Kaplan-Meier product limit formula; p-values for risk differences calculated from 1,000 bootstrap repetitions.

## Discussion

Direct comparison of gel and capillary electrophoresis genotyping methods for two anti-malarial trials showed that capillary electrophoresis was able to detect more alleles and provide higher discriminatory power than gel electrophoresis in a real-world setting. This finding is in line with published data using laboratory controls, showing that capillary electrophoresis is more sensitive in detecting minority alleles than gel electrophoresis and provides more precise sizing, allowing for better discrimination of alleles [7]. These advantages led to measured genotyping-adjusted risks of treatment failure that were significantly lower with capillary versus gel electrophoresis in a trial conducted in a site with high malaria transmission, but not in a site with moderate malaria transmission. A larger difference between methods is expected in a site of higher transmission intensity, since a higher proportion of subjects will be genotyped due to a higher rate of new infections and because the MOI will often be higher, increasing the risk of genotyping errors. While absolute risks of treatment failure were significantly different between the two methods at the high transmission site, comparative results were similar. Therefore, using capillary rather than gel electrophoresis is likely to result in obtaining a more accurate risk of treatment failure from anti-malarial trials performed in areas of high transmission intensity, but may not affect measures of differences in efficacy between treatment arms.

It is important to note that there were a large number of discordant results between genotyping methods even in the trial conducted in Kanungu, an area of moderate malaria transmission where final anti-malarial trial outcomes were similar (11 of 19 subjects classified as recrudescent by at least one method were discordant). The ability to conduct studies looking for associations between exposures, such as markers of parasite resistance or host factors, with the risk of treatment failure requires analysis on the level of the individual subject. Therefore, studies aiming to estimate such associations are more sensitive to genotyping errors than aggregate trial results. It had been previously shown that genotyping misclassification of a small number of subjects can results in a large difference in association studies [15]. While it is not possible to say with certainty that the outcome classifications are more accurate with capillary than gel electrophoresis in the absence of a gold standard, the higher sensitivity of detection and higher discrimination of capillary electrophoresis suggests that capillary results are more likely correct. Therefore, anti-malarial studies looking for exposures associated with treatment failure may benefit from genotyping with capillary electrophoresis even if performed in areas of moderate transmission intensity. The high degree of discordant results also serves to emphasize that there are limits to genotyping accuracy, regardless of the method used, especially when performed in high transmission areas.

Capillary electrophoresis offers practical as well as technical advantages over gel electrophoresis. Capillary electrophoresis offers higher throughput both for electrophoresis (96 samples run in about 1 hour) and reading of results, once familiar with the software. Aside from savings in labour costs, the capillary method also cost us slightly less for reagents and consumables, since some of the nested reactions can be multiplexed and all are performed with lower reaction volumes when analysed via capillary electrophoresis ($233/96 samples for MSP2 gel vs. $204/96 samples for MSP2 capillary). The major disadvantage of capillary electrophoresis is the need to have an expensive machine and software available for analysing samples. Though widely available in research institutions in the developed world, this capacity is still not locally available in many malaria endemic areas. In addition, laboratories familiar with gel electrophoresis would need to invest time in learning how to analyse electropherograms to distinguish true alleles from artefact. While this analysis can be automated for microsatellite loci, as stated by other authors [7], discrimination of alleles of polymorphic surface antigens is still best performed manually due to the inconsistent nature of artifacts. This is an area that could benefit from additional work.

## Conclusions

Capillary electrophoresis has improved sensitivity and discriminatory power compared to standard agarose gel electrophoresis and should be performed when genotyping anti-malarial trials performed in areas of high transmission intensity, particularly if an outcome of interest is the absolute risk of treatment failure. In addition, capillary electrophoresis may improve the ability to detect associations with treatment failure, even when studies are not performed in high transmission areas. In resource-limited settings, where capillary electrophoresis is not available, agarose gel electrophoresis appears adequate for analysis of comparative rates of treatment failure in moderate transmission settings.

## Competing interests

The authors declare that they have no competing interests.

## Authors' contributions

VG performed data collection, assisted with the statistical analysis, and drafted the manuscript. GD helped conceive of the study, participated in its design and coordination, and helped to draft the manuscript. AH provided guidance with the statistical analysis and helped to draft the manuscript. PR assisted with the study design and helped to draft the manuscript. BG helped conceive of the study, participated in its design, provided advice on troubleshooting of experiments, conducted the statistical analysis, and helped draft the manuscript. All authors read and approved the final manuscript. All authors contributed significantly to this work and declare no conflict of interest.

## Supplementary Material

Additional file 1 **Details of PCR primers and conditions for capillary electrophoresis protocols**. Table S1, Details of PCR primers and conditions for capillary electrophoresis protocols. Click here for file
