# Supplementary material for: Gel versus capillary electrophoresis genotyping for categorizing treatment outcomes in two anti-malarial trials in Uganda
Source: Malar J. 2010 Jan 15;9:19. doi: 10.1186/1475-2875-9-19 (PMC2817701; doi:10.1186/1475-2875-9-19)
Supplement: Additional file 1 — Details of PCR primers and conditions for capillary electrophoresis protocols. Table S1, Details of PCR primers and conditions for capillary electrophoresis protocols. [file 1475-2875-9-19-S1.DOC]

**Additional file 1, Table S1:** Details of PCR primers and conditions for capillary electrophoresis protocols

| **Locus** | **Primer description** | **Primer sequence** | **Cycling conditions** | **Master mix compostiion** |
| --- | --- | --- | --- | --- |
| *msp-2* | ^a^*Primary:*  S2 (fw)  S3 (rev) | 5’-GAA GGT AAT TAA AAC ATT GTC-3’  5’-GAG GGA TGT TGC TGC TCC ACA G-3’ | 94ºC - 5 min  94ºC – 30 sec  45ºC – 45 sec 25 cycles  70ºC - 90 sec  70ºC - 10 min | * 1x buffer  * 1.5mM MgCl  * 200μM dNTP  * 0.75 units Taq  * 500nM primer |
|  | ^b^*Nested:*  S_tail_  M5 (rev-FC27)  N5 (rev-3D7) | 5’-gtgtcttGCTTATAATATGAGTATAAGGAGAA-3’  5’-/6FAM/GCA TTG CCA GAA CTT GAA-3’  5’-/HEX/CTG AAG AGG TAC TGG TAG A-3’ | 94ºC - 5 min  94ºC – 30 sec  52ºC – 45 sec 40 cycles  70ºC - 90 sec  70ºC - 10 min | * 1x buffer  * 1.5mM MgCl  * 200μM dNTP  * 0.4 units Taq  * 40nM Stail primer  * 20nM M5 primer  * 20nM N5 primer |
| *msp1* | ^a^*Primary:*  N1-fw  N2-rev | 5’-GCAGTATTGACAGGTTATGG-3’  5’-GATTGAAAGGTATTTGAC-3’ | 94ºC - 5 min  94ºC – 30 sec  45ºC – 45 sec 30 cycles  70ºC - 90 sec  70ºC - 10 min | * 1x buffer  * 1.5mM MgCl  * 125μM dNTP  * 30nM primers  * 1 unit Taq |
|  |  |  |  |  |
|  | ^c^*Nested:*  K1 – F  K1 – R  MAD20 F  MAD20 R  RO33 F  RO33-R2 R | 5’-/VIC/AATGAAGAAGAAATTACTACAAAAGGTGC-3’  5’-gtgtcttGCTTGCATCAGCTGGAGGGCTTGCACCAG-3’  5’-/6FAM/AAATGAAGGAACAAGTGGAACAGCTGTTAC-3’  5’-gtgtcttATCTGAAGGATTTGTACGTCTTGAATTACC-3’  5’-/HEX/TAAAGGATGGAGCAAATACTCAAGTTGTTG-3’  5’-gtgtcttCAAGTAATTTTGAACTCTATGTTTTAAATC-3’ | 94ºC - 5 min  94ºC – 30 sec  59ºC – 45 sec 35 cycles  70ºC - 90 sec  70ºC - 10 min | * 1x buffer  * 1.5mM MgCl  * 125μM dNTP  * 200nM primers  * 1 unit Taq |
| *glurp* | ^d^*Primary:*  GF3  GF4 | 5’-ACATGCAAGTGTTGATCCTGAAG-3’  5’-gtgtcttTGTAGGTACCACGGGTTCTTGTGG-3’ | 95ºC - 4 min  94ºC – 1 min  58ºC – 2 min 30 cycles  72ºC - 2 min  70ºC - 10 min | * 1x buffer  * 2.0mM MgCl  * 200μM dNTP  * 30nM primers  * 1 unit Taq |
|  | ^d^*Nested:*  GF4  GNF | 5’-gtgtcttTGTAGGTACCACGGGTTCTTGTGG-3’  5’-/6FAM/TGTTCACACTGAACAATTAGATTTAGATCA -3’ | 95ºC - 4 min  94ºC – 1 min  61ºC – 2 min 35 cycles  72ºC - 2 min  70ºC - 10 min | * 1x buffer  * 2.0mM MgCl  * 200μM dNTP  * 40nM primers  * 1 unit Taq |

Primers derived from: ^a^ [1], ^b^ [2], ^c^ [3], ^d^ [4] and personal communication with Ingrid Felger

1. Foley M, Ranford-Cartwright LC, Babiker HA: **Rapid and simple method for isolating malaria DNA from fingerprick samples of blood**. *Mol Biochem Parasitol* 1992, **53**:241-244.

2. Falk N, Maire N, Sama W, Owusu-Agyei S, Smith T, Beck HP, Felger I: **Comparison of PCR-RFLP and Genescan-based genotyping for analyzing infection dynamics of *Plasmodium falciparum***. *Am J Trop Med Hyg* 2006, **74**:944-950.

3. Snounou G, Beck HP: **The use of PCR genotyping in the assessment of recrudescence or reinfection after antimalarial drug treatment**. *Parasitol Today* 1998, **14**:462-467.

4. Snounou G: **Genotyping of Plasmodium spp. nested PCR**. *Methods Mol Med* 2002, **72**:103-116.
